# Genetically proxied inhibition of tumor necrosis factor and the risk of colorectal cancer: A drug-target mendelian randomization study

**DOI:** 10.3389/fphar.2022.1079953

**Published:** 2022-12-23

**Authors:** Min Chen, Qian Chen, Xin-Yu Xiao, Si-Jia Feng, Xiao-Ying Wang, Tai-Chun Tang, Hui Zheng

**Affiliations:** ^1^ Department of Colorectal Diseases, Hospital of Chengdu University of Traditional Chinese Medicine, Chengdu, China; ^2^ The Third Hospital/Acupuncture and Tuina School, Chengdu University of Traditional Chinese Medicine, Chengdu, China

**Keywords:** TNF inhibition, colorectal cancer, drug target, mendelian randomization, cancer prevention

## Abstract

**Background:** Previous studies suggested that anti-TNF drugs might be repurposed as a preventive treatment for colorectal cancer. We aimed to examine whether genetically proxied inhibition of tumor necrosis factor receptor 1 (TNFR1) reduces the absolute risk of colorectal cancer through mendelian randomization (MR) analysis.

**Methods:** We obtained 28 single nucleotide polymorphisms (SNPs) that were located within a ±15 kilobase window of the TNFRSF1A—the gene that encodes the TNFR1 protein, and we used genetic data from three GWAS studies of circulating levels of TNFR1, C-reactive protein (CRP), and white blood counts (WBC) to screen SNPs that proxied the inhibition of TNFR1. Positive control analyses were then performed by using another three GWAS data from the ulcerative colitis cohort (*n* = 45,975), Crohn’s disease cohort (*n* = 40,266), and multiple sclerosis cohort (*n* = 115,803) to confirm the effect of the included SNPs. A two-sample mendelian randomization analysis was performed to examine the association between TNFR1 inhibition and the absolute risk reduction (ARR) of colorectal cancer.

**Results:** We finally included seven SNPs to proxy the anti-TNF effect, and these SNPs caused lower levels of TNFR1, CRP, and white blood counts. In positive control analyses, the included SNPs caused lower odds ratio of ulcerative colitis and Crohn’s disease but a higher odds ratio of multiple sclerosis, consistent with drug mechanistic actions and previous trial evidence. By using the inverse-variance weighted analyses to combine the effects of the seven SNPs, we found that the anti-TNF effect was associated with a 0.988 (95%CI 0.985–0.991) mg/L decrease in CRP levels and a reduction in the risk of colorectal cancer (absolute risk reduction -2.1%, 95%CI -3.8% to -0.4%, *p* = 0.01).

**Conclusion:** Our study confirmed that anti-TNF drugs were associated with a risk reduction in colorectal cancer. Physicians could consider using anti-TNF drugs for the prevention of colorectal cancer, especially in patients with high risks of developing cancer.

## Introduction

Colorectal cancer ranks third in the incidence of malignant tumors and is the second leading cause of cancer death. More than 1.85 million new colorectal cancer cases are diagnosed each year, with about 850,000 deaths globally (Biller and Schrag, 2021). Early screening and improved treatment, including fecal occult blood tests and colonoscopy, can substantially reduce morbidity and mortality. Although colonoscopy can reduce the risk of colorectal cancer and mortality, it involves high costs and medical resources, which are limited by differences in regional economic development (Keum and Giovannucci, 2019). So more affordable preventive measures become more important. Aspirin, a chemo-preventive drug, is currently the most promising drug, which can reduce the incidence of colorectal cancer by 40%. But it is not recommended for large-scale clinical use because it takes nearly 10 years to achieve a beneficial effect and increases the risk of gastrointestinal bleeding (Wilkins et al., 2018). Recently metformin, the first-line oral drug for type 2 diabetes, has also been shown to prevent adenoma recurrence (Umezawa et al., 2019). Other chemoprophylaxis, including non-steroidal anti-inflammatory drugs, cyclooxygenase-2 inhibitors, and hormone therapy, have also been reported to reduce the risk of colorectal cancer and adenomatous polyps, but they are potentially harmful and have not been widely used (Wilkins et al., 2018).

A study using infliximab to intervene in a mice model of inflammatory bowel disease (IBD) showed a reduced incidence of colorectal cancer, with only 16.7% developing colon tumors (Kim et al., 2010). In a recent animal study, TNF blockade was found to attenuate the development of colitis and colorectal cancer, mainly involving anti-inflammatory effects, reduced DNA damage response to colonic crypt regeneration, altered gut microbiota in mice, and functionally attenuated colorectal cancer development (Yang et al., 2020). In a retrospective clinical study, IBD patients treated with anti-tumor necrosis factor drugs were less likely to develop colorectal cancer (patients with Crohn’s disease: odds ratio, 0.69; 95% CI, 0.66–0.73, *p* < 0.0001; patients with ulcerative colitis: odds ratio, 0.78, 95% CI, 0.73–0.83, *p* < 0.0001) (Alkhayyat et al., 2021). These studies confirmed that the use of anti-TNF drugs in IBD patients can reduce the incidence of colorectal cancer, in addition, other disease groups also showed a decrease in colorectal cancer. A British Rheumatology Biology Society Rheumatoid Arthritis Registry study showed that patients exposed to anti-TNF therapy may have a reduced risk of colorectal cancer (hazard ratio, 0.52, 95%CI, 0.30–0.89) (Mercer et al., 2015). Based on the previous results, we found that anti-TNF drugs can reduce the incidence of colorectal cancer not only in IBD patients but also in other disease groups. Therefore, anti-TNF was chosen as the entry point of colorectal cancer prevention in our study.

There has been no randomized controlled trial that was conducted to test the efficacy of anti-TNF drugs in the prevention of colorectal cancer, leading to uncertainty about whether anti-TNF drugs are beneficial for reducing the risk of colorectal cancer. Performing a new randomized controlled trial was both time-consuming and costly. Drug-target mendelian randomization (MR) is, therefore, a new research design that is specifically developed for drug repurposing, finding new treatment targets, and unveiling the harms of drugs. The drug-target MR, in contrast to conventional MR that includes genetic instruments from the whole genome, selects the instruments (cis-variants) from the vicinity of a gene that encodes the target protein, to mimic inhibition of the gene and its encoded protein with a specific biological function (Schmidt et al., 2020). Since the genetic variances are randomly allocated at conception, the drug-target MR is analogous to a randomized controlled trial, which avoids confounding bias in observational studies.

We adopted the drug-target MR design and used data from large-scale genome-wide association studies (GWASs), to examine the impact of the genetically proxied TNF inhibition on the risk of colorectal cancer.

### Role of the funding source

The sponsors had no role in the design and conduct of the study, and they had no role in the decision process to submit the manuscript for publication.

## Methods

### Study design

We conducted a mendelian randomization analysis, using publicly available GWAS data to examine the association between genetically proxied inhibition of TNFR1 and the risk of colorectal cancer. We first determined the single nucleotide polymorphisms (SNPs) that were located in the vicinity of the gene encoding TNFR1 and had an inhibitory effect on TNFR1. We then examined whether these SNPs had effects on the downstream inflammatory biomarkers and clinical traits that are correlated to the inhibition of TNF, and we tested whether these SNPs that had inhibitory effects on TNF reduced the risk of colorectal cancer. Ethical approvals and written informed consent were acquired in each participating center. Our study adopted the summary-level genetic data and needed no additional ethical approvals. A brief description of the included GWAS studies were shown in Table 1.

**TABLE 1:** Characteristics of the included studies.

Traits	Total sample size	Cases/controls	Brief description	Main measurements	Data source
Inflammatory biomarker					
TNFR1 levels	21,758	21,758	Summary statistics from the GWAS of the proteins associated with cardiovascular diseases were obtained from 13 cohorts of European ancestry (>30,000 individuals)	Measured by using standard laboratory techniques	PMID: 33067605
CRP levels	204,402	204,402	Two GWASs were performed using data from 88 studies including individuals of European ancestry within the Cohorts for Heart and Aging Research in Genomic Epidemiology Inflammation Working Group, of circulating amounts of CRP—revealed 58 distinct genetic loci	Measured by using standard laboratory techniques, and the values were transformed by natural log (mg/L)	PMID: 30388399
WBC	172,435	172,435	A GWAS was performed in the UK Biobank and INTERVAL studies, testing genetic variants for association with 36 red cell, white cell, and platelet properties in 173,480 European-ancestry participants	Research blood samples were collected in EDTA vacutainers and measured with standard laboratory techniques	PMID: 27863252
Clinical traits					
Ulcerative colitis	45,975	12,366/33,609	A GWAS yielding a total sample size of 59,957 subjects was conducted in the International Inflammatory Bowel Disease Genetics Consortium cohort study	Clinically ascertained in the International Inflammatory Bowel Disease Genetics Consortium, and the values were presented by log odds ratio	PMID: 28067908
Crohn disease	40,266	12,194/28,072	A GWAS yielding a total sample size of 59,957 subjects was conducted in the International Inflammatory Bowel Disease Genetics Consortium cohort study	Clinically ascertained in the International Inflammatory Bowel Disease Genetics Consortium, and the values were presented by log odds ratio	PMID: 28067908
Multiple sclerosis	115,803	47,429/68,374	Genetic data of 47,429 multiple sclerosis and 68,374 control subjects were analyzed to establish a reference map of the genetic architecture of MS.	Clinically ascertained in the International Multiple Sclerosis Genetics Consortium cohort	PMID: 31604244
Colorectal cancer	377,673	5,657/372,016	The study was a cohort from the UK Biobank. Proximal colon cancers included those found within the caecum, appendix, ascending colon, hepatic flexure, transverse colon, and splenic flexure. Distal colon cancers included those found within the descending and sigmoid colon. Cancer of the rectum included cancers occurring at the recto-sigmoid junction and rectum	Clinically ascertained in the UK Biobank by using the International Classification of Diseases 10th version, and values were measured by absolute risk	UK Biobank

Abbreviation: CRP, C-reactive protein. GWAS, Genome-wide association studies. TNFR1, tumor necrosis factor receptor 1. WBC, white blood count.

### Selection of instrumental variables

To proxy TNFR1 inhibition, we first obtained 28 SNPs within ±15 kilobase windows from the gene—tumor necrosis factor receptor superfamily, member 1A (TNFRSF1A), which was located in the Chromosome 12 (base pairs: 6,328,757–6,342,114, as per GRCh38. p13 assembly) and encodes the TNFR1 protein. Secondly, we tested whether the 28 SNPs had inhibitory effects on TNFR1 by using data from three GWAS studies that determined genetic variants for the levels of TNFR1(n = 21,758) (Folkersen et al., 2020), C-reactive protein (CRP) (*n* = 204,402) (Ligthart et al., 2018), and white blood counts (WBC) (*n* = 172,435) (Astle et al., 2016). The three GWAS studies were described in detail in Table 1. From the perspective of the acknowledged physiological mechanisms, the inhibition of TNFRSF1A will cause lower levels of TNFR1, CRP—as the downstream molecular of TNF signaling, and WBC. We selected TNFR1, CRP, and WBC as the indicators of TNF inhibition, because TNFR1 was the direct target of inhibition of SNPs, which can be observed to examine the effect of the selected SNPs. In addition, CRP and WBC were the classical downstream targets of TNF signaling, and they were also used as indicators of TNF inhibition in the previously published mendelian randomization studies (Kang et al., 2021, 2022). We selected the SNPs that were associated with the decrease in the three biomarkers, and we specifically chose CRP as a proxy instrument to detect the magnitude of the inhibitory effect, because of its vital role in the TNF signaling pathway and its specific measurement in the GWAS study—measured as natural-log transforms of mg/L. The SNPs that caused reductions in all three biomarkers were included, and we used a cut-off point of *p* < 0.05 for significant associations and stringent criteria of linkage disequilibrium (r2<0.001) to further screen the SNPs, as reported in previous studies (Schmidt et al., 2020; Kang et al., 2021, 2022).

### Positive control analyses

We performed positive control analyses adopting ulcerative colitis, Crohn’s disease, and multiple sclerosis as outcomes, to validate whether the selected SNPs had a similar effect on these outcomes as the previous trials reported. Anti-TNF drugs (eg., adalimumab and infliximab) are effective treatments for IBD since they suppress inflammation in intestinal tissues and promote mucosal healing (Neurath and Travis, 2012; Singh et al., 2020; Geyl et al., 2021; Turner et al., 2021; Peyrin-Biroulet et al., 2022). We expected that the selected SNPs were associated with a reduced risk of ulcerative colitis and Crohn’s disease since these SNPs exert anti-TNF effects. Several studies reported that patients with IBD developed multiple sclerosis after they used anti-TNF drugs (Avasarala et al., 2021; Cortese et al., 2021), so we expected that the selected SNPs would be associated with a higher risk of multiple sclerosis. We selected three GWAS studies of ulcerative colitis (*n* = 45,975), Crohn’s disease (*n* = 40,266), and multiple sclerosis (*n* = 115,803) to carry out the positive control analyses, and these studies were described in detail in Table 1.

### Statistical analysis

We used CRP level as the proxy to estimate the effects of TNFR1 inhibition on the risk of colorectal cancer since CRP was theoretically the downstream molecule of TNF signaling, and the elevation of CRP level was reported to be associated with an increased level of colorectal cancer incidence in two large cohorts (Erlinger et al., 2004; Otani et al., 2006). The SNP-CRP association estimates and SNP-colorectal cancer association estimates were extracted from the GWAS studies, merged into one dataset, and harmonized for the direction of effect. We estimated the causal effect of each SNP on the risk of colorectal cancer by using the Wald ratio method. The overall effect of the selected SNPs in combination was estimated by using a fixed-effects inverse-variance-weighted meta-analysis. We performed the Cochran’s Q test to detect any potential heterogeneity in the SNP effects and any types of pleiotropy, using a cut-off point of *p* < 0.05 for the existence of heterogeneity or pleiotropy. Compared with the conventional MR design, the drug-target MR design that we used in this study is less prone to horizontal pleiotropy (Schmidt et al., 2020), so the analysis of Mendelian Randomization Pleiotropy RESidual Sum and Outlier (MR-PRESSO) was not performed. We performed two sensitivity analyses—weighted median analysis and MR Egger analysis.

To ensure that the risk-reduction effect on colorectal cancer was mediated through the inhibition of TNFR1 instead of through genetic colocalization of CRP and colorectal cancer, we performed colocalization analysis to calculate the posterior probability for the following hypothesis: 1) the association with CRP and colorectal cancer was linked in two independent SNPs; 2) the association of them was linked in one shared SNP. We adopted a Bayesian framework to estimate PP1 and PP2(Giambartolomei et al., 2014), which can accurately estimate the probability of colocalization with only GWAS summary-level data.

The MR analysis and colocalization analysis were performed in the R version 4.1.1, with the packages Twosamplesize 0.5.6 and coloc 5.1.0.1.

## Results

### Selection of instrument variables

**FIGURE 1:**
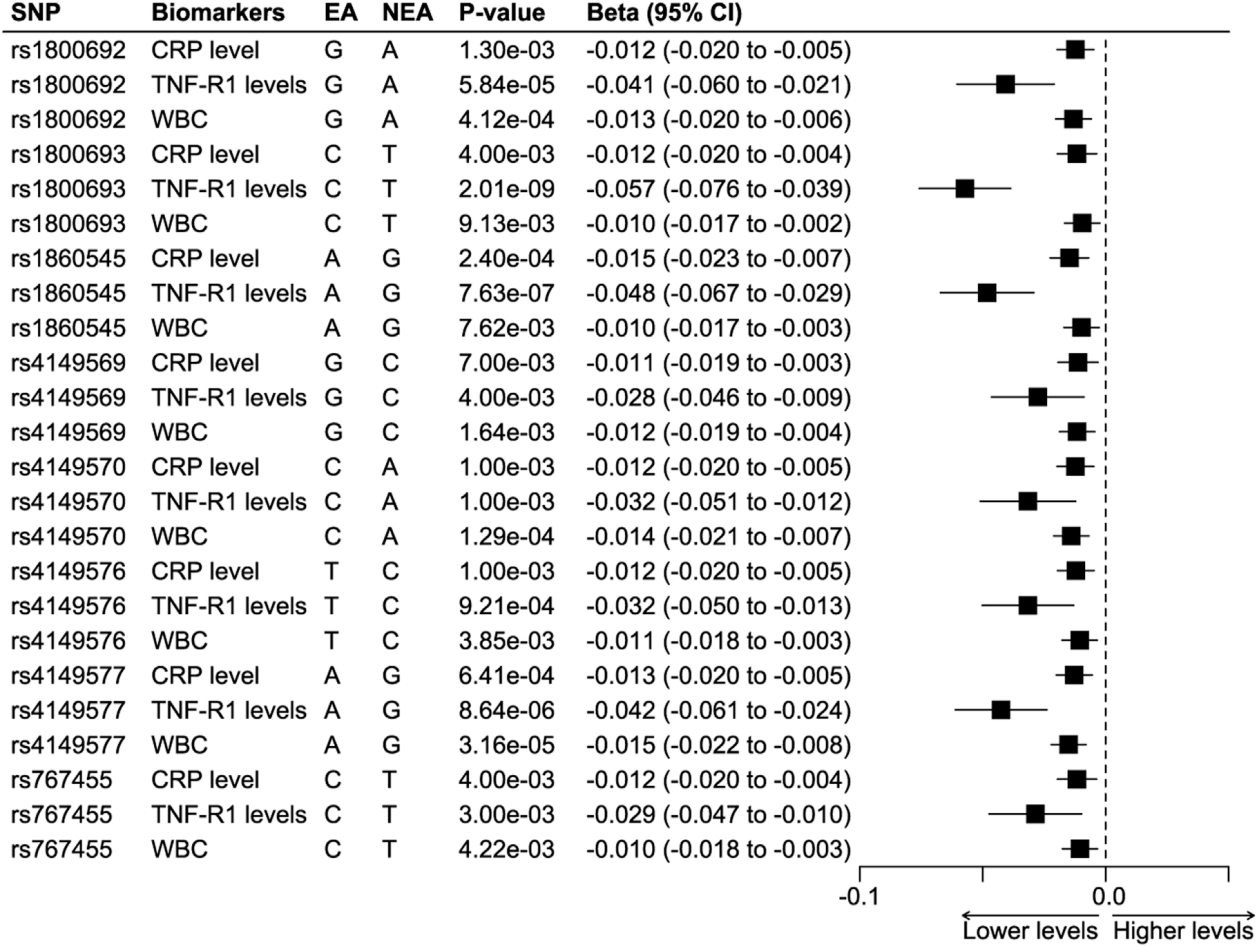
The association estimates of the eight SNPs with inflammatory biomarkers. Abbreviations: CRP, C-reactive protein. EA, effect allele. NEA, non-effect allele. SNP, single nucleotide polymorphism. TNFR1, tumor necrosis factor receptor 1. WBC, white blood count. Footnote: The results showed that all the SNPs were associated with statistically significant lower levels of TNFR1, CRP, and WBC.

Among the 28 SNPs in the TNFRSF1A gene vicinity, 15 had at least one association estimate with one of the six traits. Seven out of the 15 SNPs were excluded for violation of the inclusion criteria, which indicates a low probability of these SNPs having the cis-TNF effect. Eight SNPs had consistent effects on TNFR1 and were associated with lower levels of all three biomarkers (Figure 1). The information on the 28 SNPs was shown in Supplementary Table S1.

### Positive control analyses

**FIGURE 2:**
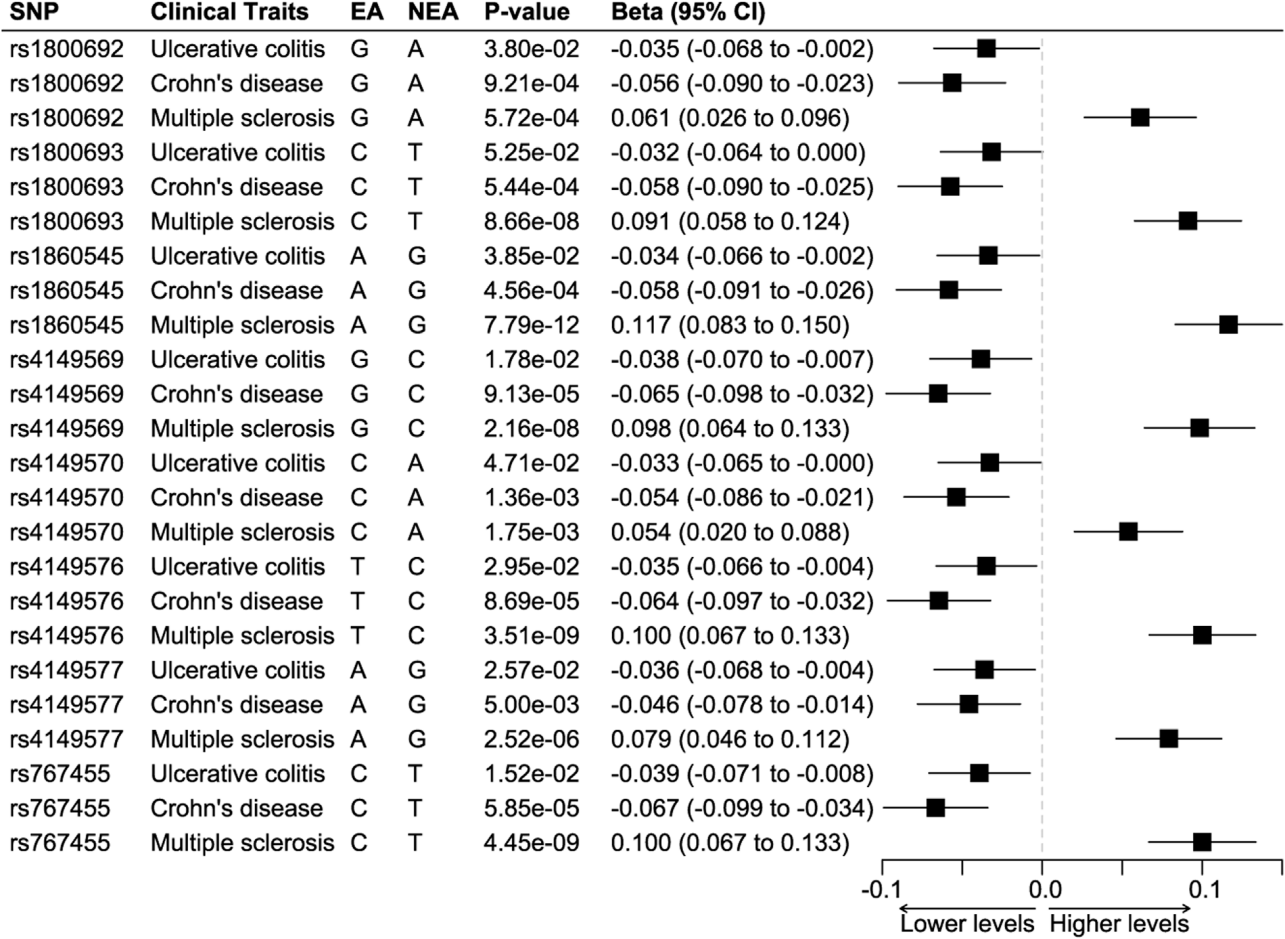
Positive control analyses. Abbreviations: EA, effect allele. NEA, non-effect allele. SNP, single nucleotide polymorphism. Footnotes: The results showed that all SNPs were associated with significantly lower odds of ulcerative colitis and Crohn’s disease but higher odds of multiple sclerosis.

The positive control analyses showed that all eight SNPs had protective effects on ulcerative colitis and Crohn’s disease while having detrimental effects on multiple sclerosis (Figure 2). We combined the effects of seven SNPs on the three diseases, since one SNP, rs4149569, was palindromic with intermediate allele frequencies. The synthesized effect of the SNPs caused a significantly lower odds ratio of ulcerative colitis (odds ratio 0.96, 95%CI 0.95 to 0.97; *p* < 0.001) and Crohn’s disease (odds ratio 0.95, 95%CI 0.94 to 0.96; *p* < 0.001) and caused significantly higher odds ratio of multiple sclerosis (odds ratio 1.09, 95%CI 1.08 to 1.11; *p* < 0.001).

### Construction and verification of cis-variants instrument

**FIGURE 3:**
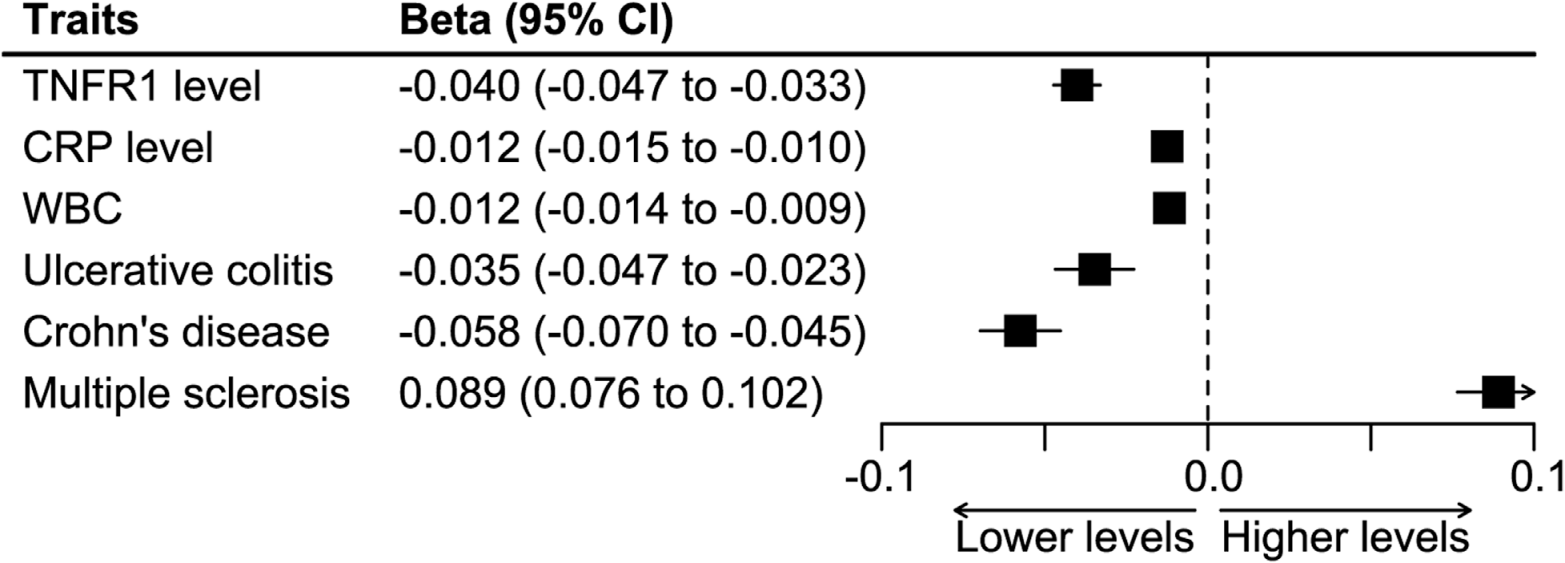
Synthesized effect of the SNPs on inflammatory biomarkers and clinical traits. Abbreviations: CRP, C-reactive protein. TNFR1, tumor necrosis factor receptor 1. WBC, white blood count.

In harmonizing the SNPs-CRP association estimates and the SNPs-colorectal cancer estimates, we excluded the SNP rs4149569 for being palindromic with intermediate allele frequencies. We further constructed the cis-variants instrument by combining the rest seven SNPs. A fixed-effects IVW meta-analysis was used in the construction, and the instrument caused lower levels of TNFR1, CRP, and WBC, and it had a protective effect on ulcerative colitis and Crohn’s disease and a detrimental effect on multiple sclerosis (Figure 3). The analysis showed that the instrument caused a decrease of 0.988 (95%CI 0.985–0.991) mg/L in the CRP level.

### TNF-inhibition and risk reduction in colorectal cancer

**FIGURE 4:**
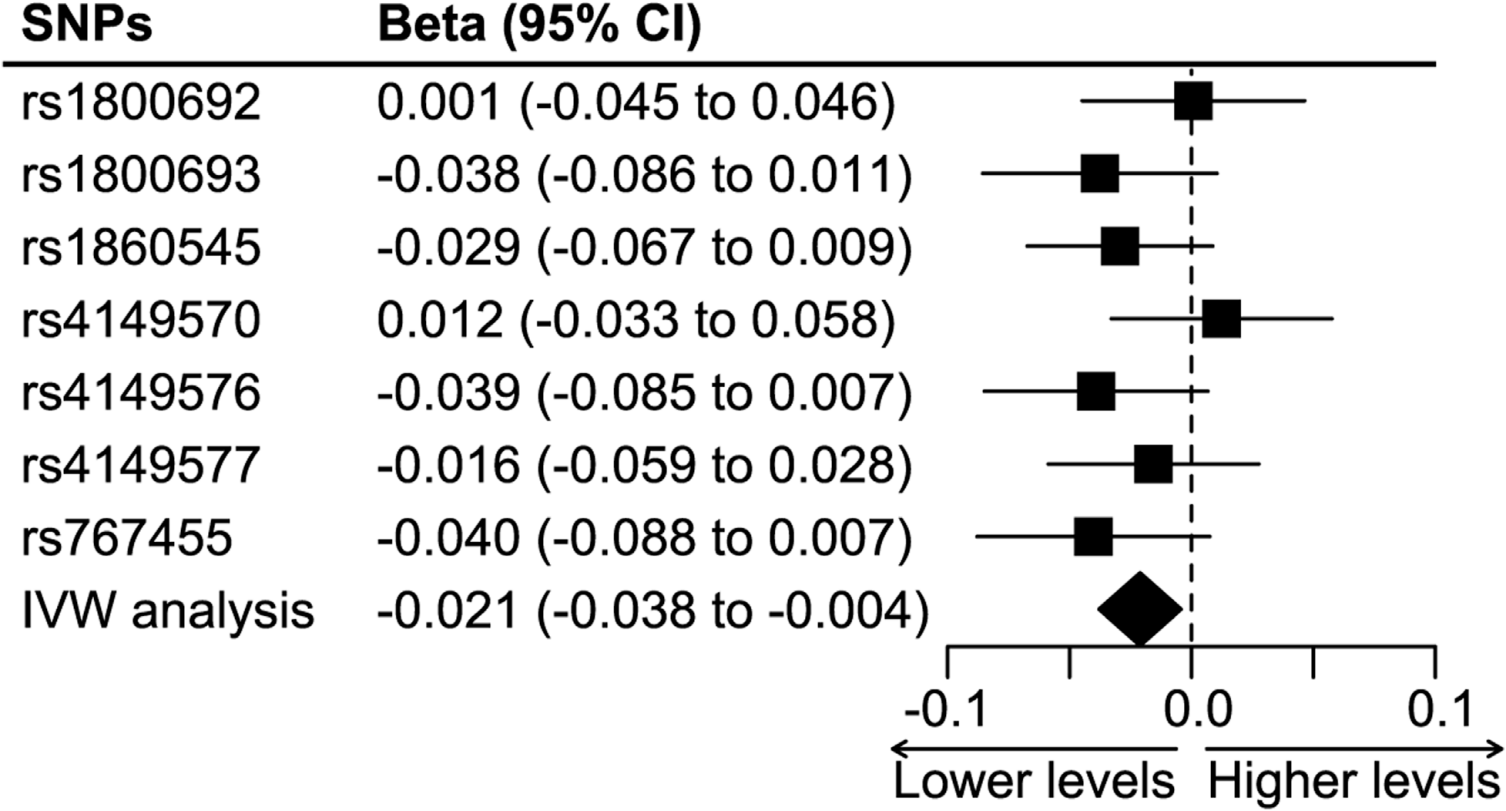
The effect of TNF-inhibition on the risk of colorectal cancer. Abbreviations: IVW analysis, inverse-variance weighted analysis. SNP, single nucleotide polymorphism. Footnotes: The IVW analysis showed a significant protective effect of TNF inhibition on colorectal cancer, and it was associated with an absolute risk reduction of 2.1% (95%CI 0.4% to 3.8%) per 1-mg/L reduction in CRP level.

Figure 4 shows the synthesized effect of TNF inhibition on colorectal cancer. The instrument of TNF-inhibition was associated with a significantly lower risk of colorectal cancer, and the results showed an absolute risk reduction of 2.1% (the IVW analysis, 95%CI 0.4%–3.8%, *p* = 0.012) per 1 mg/L reduction in CRP. The weighted median analysis showed a result consistent with the IVW analysis (absolute risk reduction 2.4%, 95%CI 0.1%–4.7%, *p* = 0.039), but the MR Egger analysis did not (absolute risk reduction 1%, 95%CI -22.9%–20.8%, *p* = 0.93).

### Colocalization analysis

We performed colocalization analysis for CRP levels and colorectal cancer within ±15 kilobase pairs of the TNRSF1A gene that encodes TNFR1 protein. We did not observe evidence for the two traits sharing two independent SNPs (posterior probability 1.12 × 10^–8^), and there was no evidence for sharing one common SNP (posterior probability 1.57 × 10^–6^).

## Discussion

In this drug-target MR analysis, we selected eight SNPs out of the vicinity of the TNFRSF1A that encodes the TNFR1 protein to mimic the inhibition of TNF, and we validated the effects of these SNPs on lowering the levels of TNFR1, CRP, and WBC. We further performed positive control analyses to examine the effect of the eight SNPs on ulcerative colitis, Crohn’s disease, and multiple sclerosis, and we found they reduced the odds ratio of developing ulcerative colitis and Crohn’s disease and increasing the odds ratio of developing multiple sclerosis, consistent with previously published studies. By using these cis-variants as proxies for TNF inhibition, we finally found that TNF inhibition was associated with a 2.1% absolute risk reduction in colorectal cancer per 1 mg/L decrease in CRP.

Evidence from previously published studies was inconsistent in whether anti-TNF drugs reduce the risk of colorectal cancer. A cohort study recruiting 734 infliximab- and 666 non-infliximab-treated patients with IBD showed that eight out of the 666 patients (1.2%) had a newly diagnosed colorectal cancer, but no infliximab-treated patients (0%) did (Biancone et al., 2009; Fidder et al., 2009). In another study of 173 IBD-associated colorectal cancer patients from 78 hospitals, anti-TNF therapy was associated with a significant protective effect against CRC development (OR, 0.09, 95% CI, 0.01–0.68, *p* = 0.02) (Baars et al., 2011). A recent cohort recruiting 225,090 individuals with Crohn’s disease and 188,420 with ulcerative colitis showed that patients treated with anti-TNF agents were less likely to develop colorectal cancer (Alkhayyat et al., 2021). These studies indicated a potentially preventive effect of infliximab on colorectal cancer. However, other studies showed contradictory results. A Danish cohort study of 35,908 IBD patients recorded in the national patient Registry between 1997 and 2015 found that UC patients who received any medical treatment (including anti-TNF) had a higher risk of CRC than UC patients who did not receive medical treatment (HR, 1.35; 95%CI, 1.01–1.81), but this was not observed in patients with CD (Weimers et al., 2021). Another study found that the incidence of CRC decreased in the RA group, while in the anti-TNF group, in contrast to other RA groups, no reduction in colorectal cancer was observed (Askling et al., 2005). Regarding the nature of observational design in these studies, these results might be affected by confounding factors. Our study, which adopted the mendelian randomization design—utilizing the random allocation of effect alleles, supported that genetically proxied TNF inhibition reduced the risk of colorectal cancer. This finding might not only apply to patients with IBD but also apply to the general population since the population we selected in the study was limited to patients with IBD.

Chronic inflammation, usually triggered by infections, aberrant immune reactions, or environmental factors, significantly increases the risk of cancer, especially colorectal cancer (Greten and Grivennikov, 2019). TNFR signaling, the major downstream of the activation of nuclear factor-κB controls the central tumor-initiating and tumor-promoting process in colorectal cancer. The TNF controls the expression of DNA methyltransferases DNA methyltransferase 1 (DNMT1) and DNMT3, leading to changes in NOTCH signaling or p53 signaling that involves in colorectal tumorigenesis (Schmitt and Greten, 2021). Blockage of TNFR1 would, therefore, reverse this biological process and have a protective effect on colorectal tumorigenesis.

We selected the level of CRP as a biomarker to measure the effect size of TNF inhibition on the risk of colorectal cancer, instead of the level of TNFR1, based on previously published literature (Kang et al., 2021, 2022). The rationale for this selection was that CRP is the downstream molecular of TNF signaling and is widely measured as a biomarker for inflammation in routine practice. One study examining the effectiveness of infliximab in the treatment of Crohn’s disease showed that, after induction of three doses of infliximab for 6 weeks, the level of CRP decreased significantly in 136 responders—the CRP level changed from 18.4 mg/L before treatment to 2.5 mg/L (IQR 2.5–8.2) after treatment, showing a decrease of nearly 16 mg/L in average (Sprakes et al., 2012). According to our study result, the TNF inhibition was associated with a 2.1% absolute risk reduction per 1 mg/L CRP, and the absolute risk reduction in this group of patients would be larger than 30% after routine infliximab treatment. Another study found similar results, the average level of CRP in patients with Crohn’s disease decreased by 13.9 mg/L at week 14 compared with baseline, and the level of decrease in CRP was maintained at 1-year follow-up lower with a treatment scheme of a 6-week induction and maintenance treatments for every 8 weeks (Gonczi et al., 2017). The change in CRP was the same in patients with ulcerative colitis (Gonczi et al., 2017).

It is worth noting that the MR Egger regression analysis provided a result that was not consistent with the IVW analysis and the weighted median analysis. First, it could be the consequence of an insufficient number of included SNPs, which might cause inaccurate estimates in the MR regression model that only works well in a large number of genetic variants (Bowden et al., 2015). Second, this finding might indicate some violations of the standard instrumental variable assumptions, which could only be tested in future interventional studies.

As the most widely used biologics in clinical practice, anti-TNF drugs not only reduced the risk of colorectal cancer in patients with IBD but also reduced the risk of colorectal cancer in patients with rheumatoid arthritis (with an adjusted hazard ratio of 0.51) (Mercer et al., 2015). The evidence from previously published studies and our study supported that the utilization of anti-TNF drugs might be a potential chemoprevention method for colorectal cancer. However, before the translation of this knowledge into practice, several questions should be further addressed. First, the target population should be determined. IBD has been proven to be a risk factor for CRC, especially in patients with ulcerative colitis, whose risk of colorectal cancer is estimated to be 2.4 times higher than that of the general population (95%CI 2.1–2.7) (Keum and Giovannucci, 2019). It is reasonable to first aim at this population. Second, the drugs and names should be further studied. Several drugs, like infliximab and adalimumab, are classified as anti-TNF, and which of these should be selected needs further clarification.

Our study had several limitations. First, the drug-target mendelian randomization design could only determine whether TNF inhibition had a protective effect on colorectal cancer. It could not provide information on which drug works better than the others. Second, the dose of anti-TNF drugs was not studied. Third, the mendelian randomization analysis studies the lifetime effect of anti-TNF on the risk of colorectal cancer, it might underestimate the real effect of anti-TNF drugs.

In conclusion, our drug-target mendelian randomization study showed a protective effect of anti-TNF on colorectal cancer. However, the target population that should receive anti-TNF for reducing the risk of colorectal cancer, the selection of a specific drug, and the treatment dose should be further investigated.

## Research in context

### Evidence before this study

We searched Pubmed and Web of Science from the database inception to 1 March 2021, for studies published in English or Chinese investigating the associations between tumor necrosis factor (TNF) levels or TNF inhibition and the risk of colorectal cancer. We used the search terms “TNF”, “TNF receptor”, “TNFR1”, “TNF inhibition”, “TNF monoclonal antibodies”, and “colorectal cancer”. Animal studies found that TNF blockade attenuated the development of colitis and colorectal cancer. Retrospective studies showed that TNF inhibition medications decreased the risk of colorectal cancer in patients with inflammatory bowel disease or rheumatoid arthritis. No randomized controlled trials or prospective observational studies were performed to study the association between TNF inhibition and the risk of colorectal cancer.

### The added value of this study

To our knowledge, this study was the first to assess whether TNF inhibition was associated with a risk reduction in colorectal cancer using a drug-target mendelian randomization design, which accelerates drug repurposing and clinical translation. We found that the anti-TNF effect was associated with an absolute risk reduction in the risk of colorectal cancer (-2.1%, 95%CI -3.8% to -0.4%, *p* = 0.01) per 1 mg/L reduction in serum C-reactive protein.

## Implications of all the available evidence

Our study finding was consistent with the previous studies, which suggested a protective effect of TNF inhibition on colorectal cancer. The drug-target mendelian randomization design selects genetic instruments—the single nucleotide polymorphisms (SNPs)—from the vicinity of the TNFR1 gene, instead of from the whole genome, which mimics the effects of the TNF-alpha antibodies (ie, adalimumab) and therefore promotes the clinical translation of the TNF-alpha antibodies into a chemoprevention modality for colorectal cancer. Although promising, the study result should be further examined in randomized controlled trials. Evidence from Mendelian randomization should be interpreted as the effect of lifetime exposure to TNF inhibition, so the dose and the time of administration should be further investigated.

## Data Availability

The original contributions presented in the study are included in the article/Supplementary Material further inquiries can be directed to the corresponding author.
